# Validation of a measure of hospital maternal level of care for the United States

**DOI:** 10.1186/s12913-024-10754-1

**Published:** 2024-03-06

**Authors:** Jennifer Vanderlaan, Jay J. Shen, Ian K. McDonough

**Affiliations:** 1 University of Nevada Las Vegas School of Nursing, 4505 S. Maryland Parkway, Box 453018, Las Vegas, Nevada 89154 USA; 2 School of Public Health, Center for Health Disparities and Research, University of Nevada Las Vegas, 4700 S. Maryland Parkway Suite #335, Las Vegas, Nevada 89154 USA; 3 Department of Economics, Lee Business School, University of Nevada Las Vegas, 4505 S. Maryland Parkway, Box 6005, Las Vegas, Nevada 89154 USA

**Keywords:** Maternal health services, Maternity hospitals, Regionalization, Supply and distribution

## Abstract

**Background:**

Lack of a validated assessment of maternal risk-appropriate care for use in population data has prevented the existing literature from quantifying the benefit of maternal risk-appropriate care. The objective of this study was to develop a measure of hospital maternal levels of care based on the resources available at the hospital, using existing data available to researchers.

**Methods:**

This was a secondary data analysis. The sample was abstracted from the American Hospital Association Annual Survey Database for 2018. Eligibility was limited to short-term acute general hospitals that reported providing maternity services as measured by hospital reporting of an obstetric service level, obstetric services, or birthing rooms. We aligned variables in the database with the ACOG criteria for each maternal level of care, then built models that used the variables to measure the maternal level of care. In each iteration, the distribution of hospitals was compared to the distribution in the CDC Levels of Care Assessment Tool Validation Pilot, assessing agreement with the Wilson Score for proportions for each level of care. Results were compared to hospital self-report in the database and measurement reported with another published method.

**Results:**

The sample included 2,351 hospitals. AHA variables were available to measure resources that align with ACOG Levels 1, 2, and 3. Overall, 1219 (51.9%) of hospitals reported resources aligned with Maternal Level One, 816 (34.7%) aligned with maternal level two, and 202 (8.6%) aligned with maternal level Three. This method overestimates the prevalence of hospitals with maternal level one compared to the CDC measurement of 36.1% (Mean 52.9%; 95% CI47.2%—58.7%), and likely includes hospitals that would not qualify as level one if all resources required by the ACOG guidelines could be assessed. This method underestimates the prevalence of hospitals with maternal critical care services (Level 3 or 4) compared to CDC measure of 12.1% (Mean 8.1%; 95%CI 6.2% – 10.0%) but is an improvement over hospital self-report (24.7%) and a prior published method (32.3%).

**Conclusions:**

This method of measuring maternal level of care allows researchers to investigate the value of perinatal regionalization, risk-appropriate care, and hospital differences among the three levels of care. This study identified potential changes to the American Hospital Association Annual Survey that would improve identification of maternal levels of care for research.

**Supplementary Information:**

The online version contains supplementary material available at 10.1186/s12913-024-10754-1.

## Background

In the United States, severe maternal morbidity occurs in 1.1 – 1.4% of inpatient deliveries [1, 2]. Severe maternal morbidity increases maternal and infant care costs and is associated with an increased risk for neonatal mortality [3–5]. Women from racialized groups experience higher rates of severe maternal morbidity [6–10]. Most strategies for preventing severe maternal morbidity are focused on ensuring quality care once the woman arrives at the hospital for birth [11, 12]. One strategy, risk-appropriate care, prevents severe maternal morbidity by ensuring the woman reaches a facility with the resources necessary to provide care commensurate with the clinical complexity of the pregnancy [13].

The purpose of risk-appropriate care is highlighted in the Three Delays Framework which hypothesizes that maternal death is the cumulative result of a lack of timely care [14]. According to the framework, there are three points where delay threatens a woman’s health to the extent that death may not be preventable, even when appropriate care is eventually provided. These points are seeking care, reaching an appropriate health facility, and receiving adequate care at the facility. Risk-appropriate care prevents the second delay in the process, reaching an appropriate health facility. In the United States, delay in reaching the appropriate health facility may be due to failure to identify the patient’s high-risk status, inequitable distribution of critical care services, or structural systems that discourage or limit transfers of care [15, 16]. Both delays in identifying a patient as high risk and failure to transfer to a higher level of care have been found to contribute to potentially preventable maternal morbidity and mortality in the U.S. and other developed countries [15, 17, 18].

The existing literature has been unable to quantify the reduction in severe maternal morbidity that can be attributed to maternal risk-appropriate care [19, 20]. The main limitation to measuring the benefits of risk-appropriate care is the lack of a validated assessment of maternal level of care in population data. Prior studies measured risk-appropriate care using a hospital self-reported maternal level of care, but were unable to measure reduction in severe maternal morbidity [19, 20]. Subsequent evidence demonstrated that hospitals over-estimate their maternal level of care, likely resulting in misclassification bias when used to evaluate outcomes with risk-appropriate care [21].

Definitions for maternal levels of care in the U.S. were published in 2015 and updated in 2019 [22–25]. The Centers for Disease Control and Prevention developed an assessment tool that aligns with the definitions of maternal level of care, but the tool has not been implemented in all states and the data is not available for research [26]. To date, two attempts have been made to measure maternal levels of care using population data [27, 28]. Both methods used the types of patients a hospital serves to identify levels of care rather than restricting measurement to the resources available at the hospitals. This means they are measuring where women at high-risk currently give birth instead of measuring the hospitals’ resources for providing complex care. This is a problem when 43% of hospitals overestimate their ability to care for complex patients [26].

The objective of this study was to develop a measure of hospital maternal levels of care based on the resources available at the hospital, using existing data available to researchers. Creation of a measure for hospital level of care for research would advance science by allowing population-level analysis of the barriers to risk-appropriate and conditions that are most effectively treated at facilities with critical care services.

## Methods

This was a cross-sectional study. The sample used to build the model was the American Hospital Association (AHA) Annual Survey Database for 2018 [29]. This year was chosen to align with the data from the CDC LOCATe Verification Pilot which was used to validate the model. Closure of hospital obstetric units since 2018 may mean more recent versions of the AHA Database are no longer representative of the sample available during the CDC LOCATe Verification Pilot. The unit of analysis was the hospital. Hospitals in the database were included in the study if they reported the hospital had an obstetric service (OBHOS = 1) or reported one or more dedicated obstetric beds (OBBD > 0). Hospitals were excluded from the study if they reported 0 births or did not report the number of births.

### Creation of measure

We began with a review of the AHA Annual Survey data dictionary to identify all variables that potentially aligned with the ACOG/SMFM criteria [24]. We created frequency tables for variables and removed from consideration any that could not be used due to low response. We organized the remaining variables according to ACOG/SMFM criteria to identify which variables had the potential to identify each level.

We worked through multiple iterations of the model validating each iteration by measuring the agreement between the distribution of hospital maternal levels of care achieved with the model to the distribution achieved in the CDC LOCATe Verification Pilot [26]. The CDC LOCATe Verification Pilot was selected as the gold standard because the survey was created to measure the resources needed for each level of care as defined by the ACOG/SMFM criteria.

Although agreement at the individual hospital level would be the most rigorous method for validation, individual hospital levels of care are not available from the CDC LOCATe Verification. Therefore, the agreement of the distribution of hospital levels of care was measured. Agreement was measured with the Wilson Score of Proportions to calculate the probability to obtaining the CDC Verification Pilot result if our measurement of the AHA data accurately represented the nation [30]. We also measured the agreement in the proportion of hospitals that self-reported a higher level than was calculated with assessment of resources using the self-reported maternal level of care in the AHA database [26].

## Results

The American Hospital Association (AHA) database included information for 6,218 hospitals, of which 2331 (37.5%) were identified as eligible for inclusion in this study. The sampling method excluded 558 hospitals that reported births, but did not report obstetric services or obstetric beds (Mean Births 740 Standard Deviation 841.6 Range 1 – 9,264). None of the excluded hospitals reported the number of operating rooms or if the hospital had ultrasound services, which are used to identify hospitals with level 1 services. Therefore, a level of care could not be measured to compare excluded hospitals to the included hospitals.

The team identified 137 variables with potential to align with resources that measure maternal level of care. After review of the missingness of these variables, the team selected 32 variables with the potential to identify resources at the hospital. The variables considered are included in Table 1, with the variable name, description, frequency and final inclusion or exclusion decision. Of these, seventeen variables had the potential to provide information that identified nine of the resources used to define the ACOG/SMFM maternal levels of care.

**Table 1:** Description of services as reported by hospitals with maternity services

**Variable**	**Description**	**Frequency *N* = 2331**	**Missing**	**Decision**	**Rationale**
ACARDHOS	Hospital has adult cardiology services	1775 (76.2%)	0	Level II	Identifies cardiologists available daily to read echocardiogram
ADTCHOS	Hospital has adult cardiac surgery	916 (39.3%)	0	Level I	Identifies basic surgical facilities for cesarean birth
CICBD	Cardiac intensive care beds	726 (31.1%)	0	Exclude	Does not add information; use MSICBD instead
CICHOS	Hospital has cardiac intensive care service	880 (37.8%)	0	Exclude	Does not add information; use MSICBD instead
CLSCIC	Hospital has a closed cardiac intensive care unit	81 (3.4%)	2266	Level III	Identifies on-sight cardiologist to interpret echocardiogram
CTSCNHOS	C-T Scanner at hospital	2305 (98.9%)	0	Level II	Identifies presence of CT Scanner
EMDEPHOS	Hospital has an emergency department	2301 (98.7%)	0	Exclude	Does not differentiate availability of services
FTECIC	Full-time cardiology intensivists	256 (11.0%)	1583	Level III	Identifies on-sight cardiologist to interpret echocardiogram
FTEMSI	Full-time medical-surgical intensivists	1060 (45.5%)	1202	Exclude	Does not add information; use MSICBD & MSICHOS
FTLAB	Number of full-time laboratory Technicians	2143 (91.9%)	0	Level I	Identifies laboratory services are readily available on-site
FTRAD	Number of full-time radiology technicians	2,271 (97.4%)	0	Exclude	Does not differentiate availability of services
HARTHOS	Hospital has heart transplant services	93 (4.0%)	0	Exclude	Does not effectively discriminate between levels III and IV
HEMOHOS	Hospital has Nephrology Services	1042 (44.7%)	0	Level III	Identifies availability of subspecialists
HSPTL	Hospital has full-time hospitalists	1911 (89.8%)	202	Level II	Indicates readily available internal or family medicine physicians
ICLABHOS	Hospital has interventional cardiac catheterization	1334 (57.6%)	0	Level III	Identifies basic interventional radiology services
KDNYHOS	Hospital has kidney transplant services	169 (7.3%)	0	Exclude	Does not effectively discriminate between levels III and IV
LIVRHOS	Hospital has liver transplant services	101 (4.3%)	0	Exclude	Does not effectively discriminate between levels III and IV
LUNGHOS	Hospital has lung transplant services	60 (2.6%)	0	Exclude	Does not effectively discriminate between levels III and IV
MAPP2	Hospital is Accredited by the American College of Surgeons Commission on Cancer	1292 (55.4%)	0	Exclude	Not specific for interventional radiology services
MSICBD	Hospital has medical-surgical intensive care beds	2222 (95.3%)	0	Level III	Identifies hospitals with medical-surgical intensive care
MRIHOS	MRI at the hospital	2120 (91.0%)	0	Level II	Identifies presence of MRI
MSICHOS	Hospital has medical-surgical intensive care service	2071 (88.9%)	0	Level III	Identifies hospitals with medical-surgical intensive care
NEROHOS	Hospital has neurology services	1607 (68.9%)	0	Level III	Identifies availability of subspecialists
ONCOLHOS	Hospital has oncology services	1780 (76.4%)	0	Level III	Identifies availability of subspecialists
OPRA	Number of operating rooms (greater than zero)	2254 (96.7%)	76	Level I	Identifies basic surgical facilities for cesarean birth
OTBONHOS	Hospital has bone marrow transplant services	134 (5.8%)	0	Exclude	Does not effectively discriminate between levels III and IV
OTHICBD	Hospital has other intensive care beds	348 (14.9%)	0	Exclude	Poor discrimination for intensive care – includes step down units
OTHIHOS	Hospital has other intensive care service	375 (16.1%)	0	Exclude	Poor discrimination for intensive care – includes step down units
PCAHOS	Hospital has patient-controlled analgesia	2118 (90.9%)	0	Level II	Identifies full-time anesthesiology service
TISUHOS	Hospital has tissue transplant services	367 (15.7%)	0	Exclude	Does not effectively discriminate between levels III and IV
TRAUML90	Trauma Level of hospital	1249 (53.6%)	1077	Level III	Allows removal of hospitals who are unlikely to meet temporal requirements
	Level 1	225 (17.9%)			
	Level 2	331 (16.4%)			
	Level 3	470 (37.5%)			
	Level 4	228 (18.2%)			
ULTSNHOS	Hospital has ultrasound services	2294 (98.4%)	0	Level I	Identifies basic ultrasound equipment

The model underwent four iterations, which can be viewed in the supplement, prior to finalizing the fifth version of the model. The first model included eight criteria identified by fourteen variables. This model failed to discriminate between specialty and critical care resources with 46.2% of the hospitals identified as having level III services and 34.3% as having level IV services. Results of the Wilson Score for each model are available in Table 2. As only one variable was potentially aligned with level IV criteria, we determined the data was not appropriate for discriminating between level III and level IV services and would instead aim for identification of level III/IV services. In the second iteration, the team reduced the model to 15 variables to identify eight criteria. The second model over-corrected, with only 6.6% of hospitals qualifying for level III/IV services.

**Table 2:** Distribution of hospitals across maternal levels of care for model iterations

**Model**	**< Level I n (%)**	**I n (%)**	**II n (%)**	**III n (%)**	**IV n (%)**
CDC Results	13.4%	36.1%	38.4%	7.1%	5.0%
Model 1	109 (4.7%)***	553 (23.7%)***	592 (25.4%)***	278 (11.9%)**	799 (34.3%)***
**Model**	**< Level I n (%)**	**I n (%)**	**II n (%)**	**III/IV n (%)**	
Model 2	109 (4.7%)***	553 (23.7%)***	1516 (65.0%)***	153 (6.6%)***	
Model 3	292 (12.5%)	736 (31.5%)*	744 (31.9%)**	559 (24.0%)***	
Model 4	269 (11.5%)	754 (32.3%)	842 (36.1%)	466 (20.0%)***	
Final Model	269 (11.5%)	825 (35.4%)	944 (40.5%)	293 (12.6%)	

^***^ *p* < .001

^**^ *p* < .01

^*^p < .05

In the third model, the team added a measure of on-site laboratory services to improve discrimination between hospitals with less than level I and level I resources, and expanded the criterion for interventional radiology to better discriminate level II services. The third model included thirteen variables across nine categories. The distribution of hospitals indicated the third model was likely overestimating the number of hospitals with level III/IV services. The fourth model added an additional variable to identify hospitals with surgical capability and removed the expanded criterion for interventional radiology. The fourth model had fourteen variables across nine categories, but was still overestimating the proportion of hospitals with level III/IV services.

The final model is presented in Table 3. The final model included a criterion for the presence of subspecialists to identify level II services. The final model also used trauma center levels, when available, to identify hospitals that did not meet the temporal requirements for availability of providers at levels II and III. Applying trauma level criteria removed 34 hospitals from the level III/IV category. The final model included seventeen variables that identified ten criteria. The distribution of hospitals using this model aligned with the CDC reporting of distribution of hospitals as assessed by non-significant Wilson Scores for each level category.

**Table 3:** Alignment of Final Model Variables with Maternal Levels of Care Criteria

**Maternal Levels of Care Criteria**	**Operationalization as AHA Variable**	**All Hospitals**
Ability to begin emergency cesarean delivery within a time interval that best incorporates maternal and fetal risks and benefits	ORPA > 0 Operating Rooms AND ADTCHOS = 1 Adult Cardiology Surgery	2278 (97.7%)
Limited obstetric ultrasonography with interpretation readily available at all times	ULTSNHOS = 1 Ultrasound services	2294 (98.4%)
Support services readily available at all times, including laboratory testing and blood bank	FTLAB > 0 Full time lab technicians	2143 (91.9%)
Capable to implement patient safety bundles for common causes of preventable maternal morbidity	No variables available	–
Ability at all times to initiate massive transfusion protocol	No variables available	–
Stabilization and the ability to facilitate transport to a higher-level hospital when necessary	No variables available	–
Ability to initiate and sustain education and quality improvement programs	No variables available	–
**Meets Level I Criteria**		**2062 (88.5%)**
Computed tomography scan, Magnetic resonance imaging, non-obstetric ultrasound imaging, and maternal echocardiography available daily	CTSCNHOS = 1 Computed-tomography (CT) Scanner AND MIRHOS = 1 Magnetic resonance imaging (MRI) AND ACARDHOS = 1 Adult cardiology service (for echocardiography)	1683 (72.2%)
Standard obstetric ultrasound imaging with interpretation readily available at all times	No variables available	
Obstetrician-gynecologist available at all times	No variables available	
Anesthesiology readily available at all times	PCAHOS = 1 Patient controlled analgesia	2118 (90.9%)
Internal or family medicine physicians and general surgeons readily available at all times	HSPTL = 1 Hospitalists provide care	1911 (82.0%)
Remove if Trauma level 4		
**Meets Level II Criteria**		**1237 (53.1%)**
In-house availability of all blood products	No variables available	
CT, MRI, Ultrasound, and echocardiography available at all times	FTECIC > 0 Full time cardiology intensivists OR CLSCIC = 1 Closed cardiac intensive care unit	781 (33.5%)
Specialized obstetric ultrasound and fetal assessment, including doppler studies, with interpretation readily available at all times	No variables available	
Basic Interventional Radiology	ICLABHOS = 1 Interventional cardiac catheterization	1343 (57.6%)
Appropriate equipment and personnel physically present at all times	No variables available	
Onsite Medical and Surgical ICUS	MSICBD > 0 Medical-surgical intensive care beds OR MSICHOS = 1 Medical-surgical intensive care service	2071 (88.9%)
Documented mechanism to facilitate and accept maternal transfers	No variables available	
Provide outreach education and patient transfer feedback to level I and level II designated facilities	No variables available	
Provide perinatal system leadership if acting as a regional center	No variables available	
Full complement of sub-specialists	NEROHOS = 1 (Neurology Service) AND HEMOHOS = 1 (Hematology Service) AND ONCOLHOS = 1 (Oncology service)	553 (23.7%)
Remove if Trauma level 3 or Level 4	Traumal90 ≠ 3 andTraumal90 ≠ 4	- 34
**Meets Level III Criteria**		**293 (12.6%)**

Using the final model, 1219 (51.9%) of hospitals reported resources aligned with maternal level I, 816 (34.7%) aligned with maternal level II, and 202 (8.6%) aligned with maternal level III/IV. In the current study, 57 (2.4%) hospitals did not provide a self-assessment for level of care. The comparison of hospital self-reported level with the final model level revealed a discrepancy for 1176 (51.7%) hospitals, which was different from the 46.4% discrepancy reported by the CDC when compared by Wilson Score (*p* < 0.001). Of those with a level discrepancy, 789 (67.1%) self-reported a level higher than assessed, which was lower than the 89.2% reported by the CDC.

## Discussion

This study examined the feasibility of measuring hospital maternal level of care using the variables available in the American Hospital Association Annual Survey. The final model achieved a distribution of hospital levels that was similar to the CDC LOCATe Verification Pilot. The model created in this study can be used by researchers to estimate the hospital maternal level of care when studying risk-appropriate care.

This study builds on prior research into methods to estimate hospital level of maternal care by designing a method that relies only on the presence of hospital resources to define levels of care. Limiting measurement to the available resources is necessary if the levels of care are used to measure maternal risk-appropriate care [31]. Risk-appropriate care means that the patient is treated in a hospital with the resources that match the complexity of the case [31]. The CDC found that 46.4% of hospitals had a discrepancy in their self-identified maternal level of care with most over-estimating their capacity [21]. This means that classifying hospital maternal level of care based on the types of patients served or the types of care provided will cause misclassification bias. Misclassification bias is likely the reason prior examination of the effects of risk-appropriate care using hospital self-identified levels of service did not find improved outcomes at the highest-level hospitals [19, 20].

A major challenge to identifying the level of maternal care using the AHA survey is that the AHA survey has limited information about the qualifications and presence of specific staff required to discriminate between maternal levels of care. The AHA includes obstetricians in the count of primary care physicians, which prevents the survey from identifying hospitals with 24-h in-house obstetrician coverage. The survey does not provide information about maternal–fetal medicine subspecialists which is needed to distinguish level II from level III services. The inability to measure presence of obstetricians and obstetrical subspecialists may be why this study found a lower proportion of hospitals overestimating their level of service than was found in the CDC Levels of Care Validation Pilot [21] Amending the AHA Annual Survey to include a question about the number of laborists employed by the hospital may help provide the needed level of discrimination and would align with the existing questions about the number of hospitalists and intensivists employed.

The AHA Annual Survey Data allowed identification of many of the physical resources included in the maternal levels of care but could not distinguish the timeliness of care provision. For example, the survey clearly identifies which hospitals have CT Scan and MRI machines, but does not distinguish between those that can perform an immediate scan in the middle of the night from those that operate the equipment during a daily schedule. We tested variables such as presence of an emergency department or the number of full-time radiology technicians, but nearly all hospitals reported these resources preventing them from being useful measures of level of care. The one exception to this problem was immediate availability of an echocardiogram. Because the echocardiogram must be read by a cardiologist, variables that indicated the presence of cardiology intensivists, presence of a closed cardiac intensive care unit, and the number of cardiac intensivists, provided an estimate of the availability of this service.

One limitation of using the AHA to measure hospital level of care is that the variables for intensive care units and presence of health care providers do not allow researchers to distinguish between resources for maternal levels III & IV. The final decision of the team was to collapse these categories for measurement. Collapsing the two categories is likely to be sufficient for most research into risk-appropriate care because hospitals in the level III category have critical care services. Researchers whose work depends on measuring hospitals with level IV services should use a more precise measurement, preferably verified using the CDC Levels of Care Assessment Tool (LOCATe).

## Conclusions

This study demonstrated that it is possible to use the American Hospital Association Annual Survey Database to estimate the hospital maternal level of care for research. The method of measurement presented in this study will allow researchers to quantify benefits with risk-appropriate care. In addition, this study identified potential changes to the American Hospital Association Annual Survey that would improve identification of maternal levels of care for research.

### Supplementary Information

**Supplementary Material 1. **

## Data Availability

Data sharing is not applicable to this article as no datasets were generated during the current study. The data analyzed in this study, the American Hospital Association Annual Survey Database, is available for purchase from the American Hospital Association at https://www.ahadata.com/.
