# Supplementary material for: Validation of a measure of hospital maternal level of care for the United States
Source: BMC Health Serv Res. 2024 Mar 6;24:286. doi: 10.1186/s12913-024-10754-1 (PMC10916325; doi:10.1186/s12913-024-10754-1)
Supplement: Supplementary file 1 — Supplementary Material 1. [file 12913_2024_10754_MOESM1_ESM.docx]

**Supplement Table 1: Model 1 Description and Results**

| **SMFM Criteria** | **Operationalization as AHA Variable** | **Hospitals** |
| --- | --- | --- |
| Ability to begin emergency cesarean delivery within a time interval that best incorporates maternal and fetal risks and benefits | ORPA > 0 Operating Rooms | 2254 (96.7%) |
| Limited obstetric ultrasonography with interpretation readily available at all times | ULTSNHOS = 1 Ultrasound services | 2294 (98.4%) |
| Support services readily available at all times, including laboratory testing and blood bank | No variables available | -- |
| Capable to implement patient safety bundles for common causes of preventable maternal morbidity | No variables available | -- |
| Ability at all times to initiate massive transfusion protocol | No variables available | -- |
| Stabilization and the ability to facilitate transport to a higher-level hospital when necessary | No variables available | -- |
| Ability to initiate and sustain education and quality improvement programs. | No variables available | -- |
| **Meets Level I Criteria** |  | **2222**  **(95.3%)** |
| Computed tomography scan, Magnetic resonance imaging, non-obstetric ultrasound imaging, and maternal echocardiography available daily. | CTSCNHOS=1 AND MIRHOS=1  Hospital has Computed-tomography (CT) Scanner  Hospital has Magnetic resonance imaging (MRI) | 2115  (90.7%) |
| Standard obstetric ultrasound imaging with interpretation readily available at all times. | No variables available |  |
| OB-gyn available at all times | No variables available |  |
| Anesthesiology readily available at all times | PCAHOS = 1 Patient controlled analgesia | 2118  (90.9%) |
| Internal or family medicine physicians and general surgeons readily available at all times | HSPTL =1 Hospitalists provide care | 1911  (82.0%) |
| **Meets Level II Criteria** |  | **1669**  **(71.6%)** |
| In-house availability of all blood products | No variables available |  |
| CT, MRI, Ultrasound, and echocardiography available at all times | No variables available |  |
| Specialized obstetric ultrasound and fetal assessment, including doppler studies, with interpretation readily available at all times | No variables available |  |
| Basic Interventional Radiology | ICLABHOS =1 interventional cardiac catheterization | 1343  (57.6%) |
| Appropriate equipment and personnel physically present at all times. | No variables available |  |
| Onsite Medical and Surgical ICUS | MSICBD >0 Hospital has medical-surgical intensive care beds | 2022  (86.7%) |
| Documented mechanism to facilitate and accept maternal transfers | No variables available |  |
| Provide outreach education and patient transfer feedback to level I and level II designated facilities. | No variables available |  |
| Provide perinatal system leadership if acting as a regional center. | No variables available |  |
| Full complement of sub-specialists | No variables available |  |
| **Meets Level III Criteria** |  | **1077 (46.2%)** |
| On-site medical and surgical care of complex maternal conditions with the availability of critical care or ICU beds | No variables available |  |
| On-site ICU care for obstetric patients with primary or co-management by MFM team. | No variables available |  |
| Perinatal system leadership | No variables available |  |
| At least one adult subspecialty available at all times for consult. | OTBONHOS = 1 (Bone marrow transplant)  or HARTHOS = 1 (Heart Transplant)  or KDNYHOS = 1 (Kidney Transplant)  or LIVRHOS = 1 (Liver transplant)  or LUNGHOS = 1 (Lung transplant)  or TISUHOS = 1 (Tissue Transplant)  ADTCHOS =1 (Adult Cardiac Surgery) | 1059  (45.4%) |
| At least one adult subspecialty available at all times for consult. |  |  |
| **Meets Level IV Criteria** |  | **799 (34.3%)** |

**Supplement Table 2: Model 2 Description**

| **SMFM Criteria** | **Operationalization as AHA Variable** | **All Hospitals** |
| --- | --- | --- |
| Ability to begin emergency cesarean delivery within a time interval that best incorporates maternal and fetal risks and benefits | ORPA > 0 Operating Rooms | 2254 (96.7%) |
| Limited obstetric ultrasonography with interpretation readily available at all times | ULTSNHOS = 1 Ultrasound services | 2294 (98.4%) |
| Support services readily available at all times, including laboratory testing and blood bank | No variables available | -- |
| Capable to implement patient safety bundles for common causes of preventable maternal morbidity | No variables available | -- |
| Ability at all times to initiate massive transfusion protocol | No variables available | -- |
| Stabilization and the ability to facilitate transport to a higher-level hospital when necessary | No variables available | -- |
| Ability to initiate and sustain education and quality improvement programs. | No variables available | -- |
| **Meets Level I Criteria** |  | **2222**  **(95.3%)** |
| Computed tomography scan, Magnetic resonance imaging, non-obstetric ultrasound imaging, and maternal echocardiography available daily. | CTSCNHOS=1 Computed-tomography (CT) Scanner  AND MIRHOS=1 Magnetic resonance imaging (MRI) | 2115  (90.7%) |
| Standard obstetric ultrasound imaging with interpretation readily available at all times. | No variables available |  |
| OB-gyn available at all times | No variables available |  |
| Anesthesiology readily available at all times | PCAHOS = 1 Patient controlled analgesia | 2118  (90.9%) |
| Internal or family medicine physicians and general surgeons readily available at all times | HSPTL =1 Hospitalists provide care | 1911  (82.0%) |
| **Meets Level II Criteria** |  | **1669**  **(71.6%)** |
| In-house availability of all blood products | No variables available |  |
| CT, MRI, Ultrasound, and echocardiography available at all times | FTECIC >0 Full time cardiology intensivists | 256  (11.0%) |
| Specialized obstetric ultrasound and fetal assessment, including doppler studies, with interpretation readily available at all times | No variables available |  |
| Basic Interventional Radiology | ICLABHOS =1 Interventional cardiac catheterization | 1343  (57.6%) |
| Appropriate equipment and personnel physically present at all times. | No variables available |  |
| Onsite Medical and Surgical ICUS | MSICBD >0 Medical-surgical intensive care beds | 2022  (86.7%) |
| Documented mechanism to facilitate and accept maternal transfers | No variables available |  |
| Provide outreach education and patient transfer feedback to level I and level II designated facilities. | No variables available |  |
| Provide perinatal system leadership if acting as a regional center. | No variables available |  |
| Full complement of sub-specialists | ADTCHOS =1 (Adult Cardiac Service)  and NEROHOS = 1 (Neurology Service)  and HEMOHOS=1 (Hematology Service)  and ONCOLHOS=1 (Oncology service) | 553  (23.7%) |
| **Meets Level III/IV Criteria** |  | **153 (6.6%)** |

**Supplement Table 3: Model 3 Description**

| **SMFM Criteria** | **Operationalization as AHA Variable** | **All Hospitals** |
| --- | --- | --- |
| Ability to begin emergency cesarean delivery within a time interval that best incorporates maternal and fetal risks and benefits | ORPA > 0 Operating Rooms | 2254 (96.7%) |
| Limited obstetric ultrasonography with interpretation readily available at all times | ULTSNHOS = 1 Ultrasound services | 2294 (98.4%) |
| Support services readily available at all times, including laboratory testing and blood bank | FTLAB >0 Full time lab technicians | 2143 (91.9%) |
| Capable to implement patient safety bundles for common causes of preventable maternal morbidity | No variables available | -- |
| Ability at all times to initiate massive transfusion protocol | No variables available | -- |
| Stabilization and the ability to facilitate transport to a higher-level hospital when necessary | No variables available | -- |
| Ability to initiate and sustain education and quality improvement programs. | No variables available | -- |
| **Meets Level I Criteria** |  | **2039**  **(87.5%)** |
| Computed tomography scan, Magnetic resonance imaging, non-obstetric ultrasound imaging, and maternal echocardiography available daily. | CTSCNHOS=1 Computed-tomography (CT) Scanner)  AND MIRHOS=1 Magnetic resonance imaging (MRI)  AND ACARDHOS = 1 Adult cardiology service | 1683  (72.2%) |
| Standard obstetric ultrasound imaging with interpretation readily available at all times. | No variables available |  |
| OB-gyn available at all times | No variables available |  |
| Anesthesiology readily available at all times | PCAHOS = 1 Patient controlled analgesia | 2118  (90.9%) |
| Internal or family medicine physicians and general surgeons readily available at all times | HSPTL =1 Hospitalists provide care | 1911  (82.0.%) |
| **Meets Level II Criteria** |  | **1303 (55.9%)** |
| In-house availability of all blood products | No variables available |  |
| CT, MRI, Ultrasound, and echocardiography available at all times | FTECIC >0 Full time cardiology intensivists | 256  (11.0%) |
| Specialized obstetric ultrasound and fetal assessment, including doppler studies, with interpretation readily available at all times | No variables available |  |
| Basic Interventional Radiology | ICLABHOS =1 interventional cardiac catheterization  Or MAPP2 = 1 Certified cancer treatment center | 1507  (64.7%) |
| Appropriate equipment and personnel physically present at all times. | No variables available |  |
| Onsite Medical and Surgical ICUS | MSICBD >0 Medical-surgical intensive care beds  Or MSICHOS=1 Medical-surgical intensive care service | 2071  (88.9%) |
| Documented mechanism to facilitate and accept maternal transfers | No variables available |  |
| Provide outreach education and patient transfer feedback to level I and level II designated facilities. | No variables available |  |
| Provide perinatal system leadership if acting as a regional center. | No variables available |  |
| Full complement of sub-specialists | No variables available |  |
| **Meets Level III/IV Criteria** |  | **559 (24.0%)** |

**Supplement Table 4: Model 4 Description**

| **SMFM Criteria** | **Operationalization as AHA Variable** | **All Hospitals** |
| --- | --- | --- |
| Ability to begin emergency cesarean delivery within a time interval that best incorporates maternal and fetal risks and benefits | ORPA > 0 Operating rooms  or ADTCHOS=1 Adult Cardiology Surgery | 228 (97.7%) |
| Limited obstetric ultrasonography with interpretation readily available at all times | ULTSNHOS = 1Ultrasound services | 2294 (98.4%) |
| Support services readily available at all times, including laboratory testing and blood bank | FTLAB >0 Full time lab technicians | 2143 (91.9%) |
| Capable to implement patient safety bundles for common causes of preventable maternal morbidity | No variables available | -- |
| Ability at all times to initiate massive transfusion protocol | No variables available | -- |
| Stabilization and the ability to facilitate transport to a higher-level hospital when necessary | No variables available | -- |
| Ability to initiate and sustain education and quality improvement programs. | No variables available | -- |
| **Meets Level I Criteria** |  | **2062**  **(88.5%)** |
| Computed tomography scan, Magnetic resonance imaging, non-obstetric ultrasound imaging, and maternal echocardiography available daily. | CTSCNHOS=1 Computed-tomography (CT) Scanner)  AND MIRHOS=1 Magnetic resonance imaging (MRI)  AND ACARDHOS = 1 Adult cardiology service | 1683  (72.2%) |
| Standard obstetric ultrasound imaging with interpretation readily available at all times. | No variables available |  |
| OB-gyn available at all times | No variables available |  |
| Anesthesiology readily available at all times | PCAHOS = 1 Patient controlled analgesia | 2118  (90.9%) |
| Internal or family medicine physicians and general surgeons readily available at all times | HSPTL =1 Hospitalists provide care | 1911  (82.0%) |
| **Meets Level II Criteria** |  | **1308 (56.1%)** |
| In-house availability of all blood products | No variables available |  |
| CT, MRI, Ultrasound, and echocardiography available at all times | FTECIC >0 Full time cardiology intensivists  Or CLSCIC=1 Closed cardiac intensive care unit | 781  (33.5%) |
| Specialized obstetric ultrasound and fetal assessment, including doppler studies, with interpretation readily available at all times | No variables available |  |
| Basic Interventional Radiology | ICLABHOS =1 interventional cardiac catheterization  Or MAPP2 = 1 Certified cancer treatment center | 1507  (64.7%) |
| Appropriate equipment and personnel physically present at all times. | No variables available |  |
| Onsite Medical and Surgical ICUS | MSICBD >0 Medical-surgical intensive care beds  Or MSICHOS=1 Medical-surgical intensive care service | 2071  (88.9%) |
| Documented mechanism to facilitate and accept maternal transfers | No variables available |  |
| Provide outreach education and patient transfer feedback to level I and level II designated facilities. | No variables available |  |
| Provide perinatal system leadership if acting as a regional center. | No variables available |  |
| Full complement of sub-specialists | No variables available |  |
| **Meets Level III/IV Criteria** |  | **466 (20.0%)** |
